# A Randomized Controlled Simulation Trial of a Neonatal Resuscitation Digital Game Simulator for Labour and Delivery Room Staff

**DOI:** 10.3390/children11070793

**Published:** 2024-06-28

**Authors:** Christiane Bilodeau, Georg M. Schmölzer, Maria Cutumisu

**Affiliations:** 1 Department of Pediatrics, Faculty of Medicine and Dentistry, University of Alberta, Edmonton, AB T6G 2E3, Canada; cebilode@ualberta.ca; 2 Department of Educational and Counselling Psychology, Faculty of Education, McGill University, Montreal, QC H3A 1Y2, Canada; maria.cutumisu@mcgill.ca

**Keywords:** neonatal resuscitation, digital game simulator, performance, delivery room, newborn

## Abstract

Background: Healthcare providers (HCPs) working in labour and delivery rooms need to undergo regular refresher courses to maintain their neonatal resuscitation skills, which are shown to decline over time. However, due to their irregular schedules and limited time, HCPs encounter difficulties in readily accessing refresher programs. RETAIN is a digital game that simulates a delivery room to facilitate neonatal resuscitation training for HCPs. Objective: This study aims to ascertain whether participants enjoyed the RETAIN digital game simulator and whether it was at least as good as a video lecture at refreshing and maintaining participants’ neonatal resuscitation knowledge. Methods: In this randomized controlled simulation trial, *n* = 42 labour and delivery room HCPs were administered a pre-test of neonatal resuscitation knowledge using a manikin. Then, they were randomly assigned to a control or a treatment group. For 20–30 min, participants in the control group watched a neonatal resuscitation lecture video, while those in the treatment group played the RETAIN digital game simulator of neonatal resuscitation scenarios. Then, all participants were administered a post-test identical to the pre-test. Additionally, participants in the treatment group completed a survey of attitudes toward the RETAIN simulator that provided a measure of enjoyment of the RETAIN game simulator. After two months, participants were administered another post-test identical to the pre-test. Results: For the primary outcome (neonatal resuscitation performance), an analysis of variance revealed that participants significantly improved their neonatal resuscitation performance over the first two time points, with a significant decline to the third time point, the same pattern of results across conditions, and no differences between conditions. For the secondary outcome (attitudes toward RETAIN), participants in the treatment condition also reported favourable attitudes toward RETAIN. Conclusions: Labour and delivery room healthcare providers in both groups (RETAIN simulator or video lecture) significantly improved their neonatal resuscitation performance immediately following the intervention, with no group differences. The findings suggest that participants enjoyed interacting with the RETAIN digital game simulator, which provided a similar boost in performance right after use to the more traditional intervention.

## 1. Introduction

Neonatal resuscitation constitutes a series of interventions applied around the time an infant is born that aim to help stabilize the infant’s breathing and circulation [1,2,3]. The Neonatal Resuscitation Program [4] is an educational program that aims to provide individuals and teams who may be required to carry out resuscitation of newborn babies with the concepts and skills of neonatal resuscitation. The NRP also recommends that healthcare providers (HCPs) maintain effective communication strategies during neonatal resuscitation activities. NRP refresher (i.e., booster) courses are recommended every two years to maintain neonatal resuscitation knowledge and skills.

However, typical training simulations take place in specially designed simulation centres that are not always accessible or available to all HCPs, such as in the case of the COVID-19 pandemic [5]. Given the importance of correctly carrying out the neonatal resuscitation steps, researchers have been striving to find the most effective alternative methods to refresh or boost neonatal resuscitation knowledge and skills over time. Specifically, they were interested in optimal ways of retaining and maintaining neonatal resuscitation skills, such as digital game simulations [6,7].

A rich body of existing research on learning via digital games has provided evidence of learning being supported in such environments [8], as well as in medical simulations more specifically [9]. Moreover, the non-linear manner of completing tasks in digital simulation games draws on the learner’s sense of autonomy, which is a core part of the self-determination theory of motivation alongside other psychological needs, such as competence and relatedness [10]. These needs underlie individual growth and development. Moreover, enjoyment is believed to derive from the satisfaction of these three needs related to well-being [11].

### Hypotheses and Research Questions

According to prior results in the literature, we hypothesize that labour and delivery room HCPs will enjoy playing the RETAIN (i.e., REsuscitation TrAINing) digital game simulator [12,13,14,15] and that RETAIN will improve their performance in neonatal resuscitation scenarios [16]. Thus, the present study aims to ascertain whether the digital game simulation instructional method was at least as good as a more traditional alternative (video lecture) at updating and maintaining participants’ neonatal resuscitation knowledge. The study poses the following research questions: (1) What were participants’ perceptions of the RETAIN digital game simulator? (2) Is there an interaction between the instructional method and time on participants’ neonatal resuscitation performance?

## 2. Literature Review

### 2.1. Performance in the Simulators

Simulations are defined as instances of technology-rich learning environments (TREs) that may provide multiple visual and verbal learning representations [17,18] and may scaffold learners through scenarios that look and feel realistic. Thus, simulations provide learners with access to materials that are contextualized in a specific domain (e.g., medical) so that learning is more concrete and meaningful rather than abstract or isolated. Learners can practice their skills deliberately and receive immediate expert feedback that is often embedded in these environments, without the stress or risks of real-life time-sensitive situations. Importantly, simulations are useful for both individuals and teams. Specifically, members of medical teams can use simulations to learn and practice their roles when interacting with patients and other team members [17]. For example, the Deteriorating Patient mobile app enables medical students to manage patient cases using their smart phone [19]. A literature review revealed that medical simulations tend to improve learners’ performance [9]. In the context of neonatal resuscitation, it was found that standard NRP [4] training improved trainees’ knowledge and skills [20,21,22,23]. Concomitantly, a computer-based simulator also improved learners’ performance, but not significantly differently than a seminar-based instructional approach [24]. However, skills tended to decline over time [20]. An experimental study sampling third-year medical students found that the knowledge of both the control and treatment groups significantly decreased after 4 months and 8 months from the initial exposure to two instructional methods (a computerized simulator and a lecture video, respectively), despite exposure to booster training [25]. Additionally, their study found the control and treatment groups to be similar in skill levels at 8 months after the initial experiment, and no significant relationships were found in knowledge of neonatal resuscitation, skills, or confidence. Moreover, their computerized simulator and the lecture videos were equally effective in maintaining resuscitation skills of medical students. A recent experimental study compared the skill decay of *n* = 49 Filipino nursing students after watching a remote video or undergoing in-person resuscitation training [26]. Then, participants were randomly assigned to receive refresher training at 2-month intervals, either in-person or via tele-simulation, at 2 and 4 months after their initial training. McCaw and colleagues [26] found skill decay after both 2 months and 4 months of their initial training but did not find any statistical differences in neonatal resuscitation skills between the students trained in-person and via videos.

### 2.2. Attitudes toward Simulators

Moreover, simulations have also been used to support self-regulated learning (SRL) processes [27] as well as scientific inquiry and knowledge organization [28]. Digital games have been used as ways to implement more realistic simulators and to carry out assessments of performance and learning, providing both product and process data. These environments that simulate realistic learning contexts potentiate learners’ autonomy, competence, and relatedness, which are pillars of self-determination theory [10]. As a result, these environments tend to promote learner motivation [9], enjoyment [11], satisfaction [25], and preference for high-fidelity simulators [29]. Despite this, an experimental study compared neonatal resuscitation training between a high-fidelity simulator and a traditional low-fidelity equipment, and it revealed no difference in performance, non-technical skills, and stress levels [30]. Given the mixed results found in the literature, this study aims to elucidate the potential of digital game simulators to achieve the same levels of support for HCP learning and maintenance of neonatal resuscitation skills as more traditional approaches while also providing an attractive learning environment.

## 3. Method

### 3.1. Participants, Procedure, and Measurement Instruments

This randomized controlled simulation trial sampled *n* = 42 labour and delivery room HCPs. As this was a simulation trial, it was not registered on clinicaltrials.gov. No participants were excluded from the study. G*Power 3.1 was employed to determine the sample size required for a 0.80 power (i.e., β = 0.20) in a 2 × 2 mixed design ANOVA [31]. For the *F*-test and ANOVA: repeated measures, within-between interaction, large effect size *η_p_*^2^ = 0.25, a correlation *r* = 0.5 between the repeated measures, a non-sphericity correction ε = 1, α = 0.05, and β = 0.20, the power analysis yielded a sample of 10 pairs, less than half of the sample of the present study. Most participants reported completing a bachelor’s degree (*n* = 34; 80.95%), a postgraduate degree (*n* = 6; 14.29%), or a diploma, certificate, or another professional program (*n* = 2; 4.76%), as well as 12.23 months on average (SD = 8.29) since the last NRP training. They filled out a consent form according to the study protocol Pro00117011 approved by the University of Alberta’s Ethics Research Board.

First, participants filled out a survey of demographic (e.g., age, gender) and background information (e.g., professional designation), and completed a pre-test, conducted on a manikin, consisting of a neonatal resuscitation simulation scenario presented in Appendix A. The simulation of a neonatal emergency scenario was administered using a manikin at all three time points (i.e., pre-test, post-test, and post-test after two months). It assessed the steps of resuscitation (e.g., stimulation, temperature management) and respiratory support taken to save a near-term infant.

Second, participants were randomly assigned to either a control or a treatment group. Randomization was conducted using a computer-generated randomization program [32] with 1:1 randomization using variable block sizes of 2 to 4. A numbered, sealed, brown envelope was opened at each step containing the group allocation. The randomization was performed by the second author, who was not involved in participant recruitment or any study procedures. The first author recruited all participants and performed all study procedures. The second and third authors performed analysis blinded to group allocation. After statistical analyses were completed, group allocation was unblinded. Participants in the control group (*n* = 21, all females) watched a neonatal resuscitation lecture video for 20–30 min, whereas those in the treatment group (*n* = 21, of which 20 were female and 1 did not report their gender) played the RETAIN digital game simulator on the same topic for the same amount of time. The video lecture was designed by the researchers and showcased the steps of the eighth edition NRP Part 2 [4], including the initial steps and respiratory support including MR. SOPA. The lecture was standardized for the participants in the control group and customized to the labour and delivery room staff members. The treatment group participants played the RETAIN digital game simulator starting with a simulated tutorial followed by a game scenario depicting neonatal resuscitation tasks of varying degrees of difficulty.

Third, participants were asked to complete a simulation post-test focused on a scenario identical to that presented in the pre-test. Participants in the treatment condition were also asked six questions measured on a 5-point Likert-scale regarding their attitudes toward the RETAIN digital game simulator, including whether the simulation scenario was realistic, enjoyable (in both interaction and format), useful for teaching neonatal resuscitation, allowed participants to make good decisions, and had appropriate length and pacing [16].

**Figure 1:**
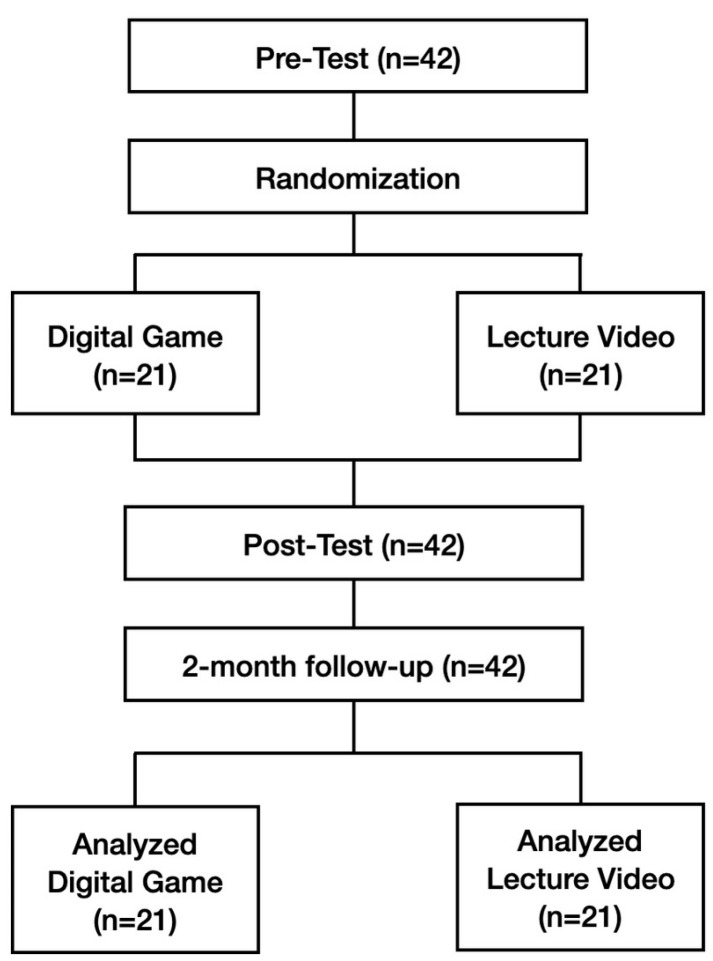
CONSORT diagram employed in this study.

Finally, two months after the intervention, participants were administered another post-test identical to the pre-test (shown in Appendix A) to ascertain whether they maintained their neonatal resuscitation performance over time following the initial training with either the RETAIN digital game simulator or the lecture video training. Figure 1 depicts the CONSORT diagram summarizing this randomized controlled simulation trial.

### 3.2. Intervention: The RETAIN Digital Game Simulator

The RETAIN digital game simulator constitutes the intervention hypothesized to affect the primary outcome of this experiment (i.e., neonatal resuscitation performance). The RETAIN digital game simulator used in the treatment condition of this experimental study was developed to simulate neonatal resuscitation scenarios of varying difficulty [12,13,14,15]. The purpose of the RETAIN digital game simulator was to provide training to HCPs regarding the execution of the steps of the official NRP Part 2 [4] algorithm as well as more opportunities to refresh their skills on their own terms. Specifically, in digital game simulations like RETAIN, HCPs can practice the steps of the NRP algorithm at their own pace, whenever they have spare time in between carrying out their daily tasks. This environment also offers HCPs the opportunity to try out different actions, especially for rarely occurring scenarios, without the stress of harming an actual infant.

When using the RETAIN digital game simulator, an HCP’s avatar is a clinical care provider whose task is to perform neonatal resuscitation activities. This digital game simulator has a tutorial that trains the player on the order and the conditions of applying neonatal resuscitation interventions as well as 50 neonatal resuscitation scenarios.

### 3.3. The RETAIN Tutorial Scenario

Players start by completing a tutorial that provides them with an opportunity to familiarize themselves with the game interface. Then, they are presented with a neonatal resuscitation scenario that requires them to perform several steps in a specific order and under certain conditions: dry the baby, assess its breathing and its colour for cyanosis, clear the airways through mouth and nose suction, improve airway function by adjusting the position of the baby’s head, apply a bag valve mask to support the baby’s breathing, apply chest compressions, endotracheal intubation, and administer epinephrine.

### 3.4. The RETAIN Game Scenarios

After completing the tutorial, the player is presented with 50 different resuscitation scenarios. In these scenarios, the player is required to assess the status of a simulated neonate (i.e., newborn baby) who experiences difficulties breathing by itself at birth. Hence, the player must provide care to the infant that is appropriate for the current state of the infant. The learner must perform the following neonatal resuscitation steps: suction, adjustment of baby’s head position, use of a bag and mask to assist breathing, compression of the baby’s chest, or endotracheal intubation and administration of epinephrine.

### 3.5. Measures

Several measures were employed in this experimental study.

*Group*. This variable represents the condition used for training (i.e., the between-subjects factor in this study). It was coded with 1 for the control condition (a lecture video on the neonatal resuscitation algorithm) and 2 for the treatment condition (a neonatal resuscitation digital game simulator).

*Time*. This variable (i.e., the within-subjects factor) consists of three time points. Performance on the neonatal resuscitation scenarios was recorded before (Time 1), immediately after the intervention (Time 2), and two months after that (Time 3).

*Performance*. This variable represents the primary outcome of this study. It measures a participant’s knowledge on the neonatal resuscitation scenarios at three time points: before the instructional intervention (*Pre-test*), right after the intervention (*Post-test*), and two months after that (*Post-test After 2 Months*), respectively. It is composed of 6 prompts equivalent to 12 steps of resuscitation (e.g., stimulation, temperature management) and respiratory support taken to save a near-term infant. Each step was marked with either 1 (correct) or 0 (incorrect), accounting for 2 points per prompt. For example, one of the prompts in the assessment scenario was the following: “The baby is transferred to the resuscitation table. What are the next steps?”. Participants were prompted for a free-form text answer to this constructed-response item worth 2 points (i.e., 1 point for each of the two steps required to answer each prompt). Thus, the performance measure at each time point ranged from 0 to 12.

*Attitudes Toward the RETAIN Simulator*. Six items regarding the attitudes of the participants regarding the RETAIN digital game simulator were collected and analysed. These variables represent the secondary outcome. They were measured on a 5-point Likert scale with the following options: 1 = Strongly Disagree, 2 = Disagree, 3 = Neutral, 4 = Agree, 5 = Strongly Agree. Thus, these measures ranged from 1 to 5. *Realistic Scenario* measures whether the RETAIN simulation scenario was realistic. *Appropriate Game Length and Pacing* measured whether the RETAIN digital game simulator had appropriate length and pacing. *Enjoyed Learning in this Game Format* measured whether participants found the game format of the RETAIN simulator enjoyable. *Enjoyed Playing the Game* captured a participant’s enjoyment of interacting with the RETAIN digital game simulator. *Game was Useful to Teach NRP Algorithm* measured whether participants found RETAIN useful to teach neonatal resuscitation. *Game Allowed Appropriate Decisions* measured whether participants appreciated that RETAIN allowed them to make good decisions within the context of neonatal resuscitation.

### 3.6. Analyses

Data analyses were conducted using R version 4.3.3 [33]. Descriptive analyses were first conducted to better understand the variables included in this experiment. An analysis of variance (ANOVA), specifically a robust two-way mixed ANOVA [34] using trimmed means from the *WRS2* [35] package (*bwtrim* function) given the two factors, a between-subjects factor of time (*sppba* function), and a within-subjects factor of group (*sppbb* function), was conducted. It aimed to ascertain if changes in participants’ performance were the result of the interaction (*sppbi* function) between the intervention (i.e., the lecture video or the digital game) and the passage of time.

## 4. Results

### 4.1. What Were Participants’ Perceptions of the RETAIN Digital Game Simulator?

First, descriptive statistics for the participants in the treatment condition are displayed in Table 1, which shows that, on average, the participants tended to enjoy the RETAIN digital game simulator and found the scenarios in the game to be realistic, with an appropriate game length and pacing. Participants also reported that the game was largely useful for teaching the NRP algorithm and allowed them to make appropriate decisions.

**Figure 2:**
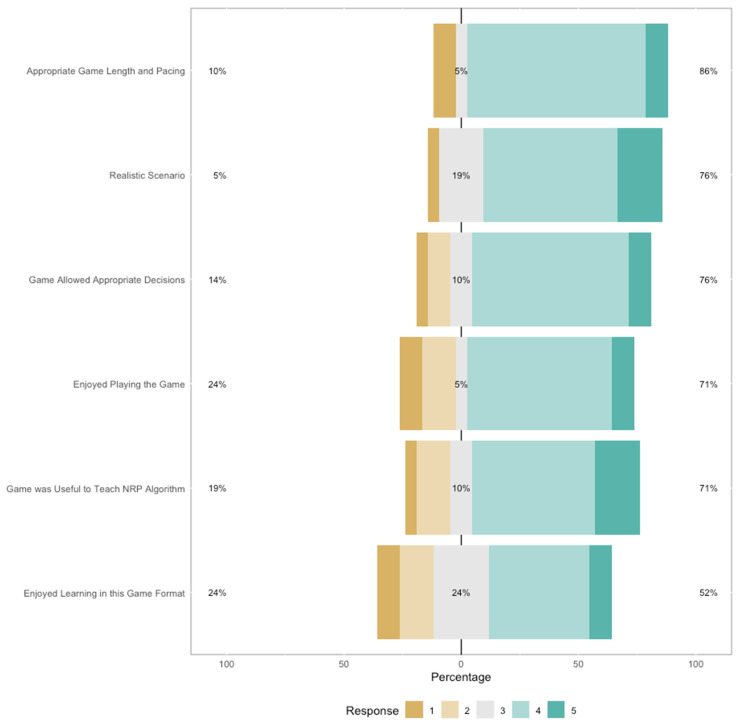
Survey responses on the game-related items.

These results are also visualized in Figure 2, which shows that, overall, most participants perceived the RETAIN digital game simulator positively. For example, participant answers to the “Enjoyed Playing the Game” item were distributed as follows: 24% strongly disagreed or disagreed with this statement, 5% were neutral, and 71% agreed or strongly agreed with this statement.

### 4.2. Is There an Interaction between the Instructional Method and Time on Participants’ Neonatal Resuscitation Performance?

First, descriptive analyses across the entire sample shown in Table 2 were conducted to examine the performance measures relevant for answering this research question. Each of the three performance measures ranged from 0 to 12 points, where a higher value indicates a better performance.

Then, before the two-way mixed ANOVA could be carried out, the assumptions for this analysis were tested. First, the data set was inspected for outliers. There was one outlier detected, but it was not excluded for two reasons: it was a valid response, and the sample size was small. Table A1 of Appendix B shows that the Shapiro–Wilk normality test was significant for all three time points in the traditional method group and for the Post-test After 2 Months of the digital game group.

For these reasons, robust two-way mixed ANOVAs on 20% trimmed means were carried out to answer the first research question. Findings revealed no *Time* × *Group* interaction predicting participants’ performance. However, Table 3 shows a statistically significantly large main effect of *Time* [*F*(2, 20.56) = 33.13, *p* < 0.001] on the neonatal resuscitation performance.

Mauchly’s tests for Time and the Group × Time interaction, respectively, are shown in Table A2, together with the sphericity corrections. Finally, findings of the two-way mixed ANOVA on the regular means are displayed in Table A3 of Appendix B.

Table 4 shows the results of the robust two-way mixed ANOVA using bootstrap with 500 bootstrap samples, including the within groups effect only (*sppbb* function), the between groups effect only (*sppba* function), and the interaction effect only (*sppbi* function), all using the modified one-step (MOM) estimator of location based on Huber’s Psi.

**Figure 3:**
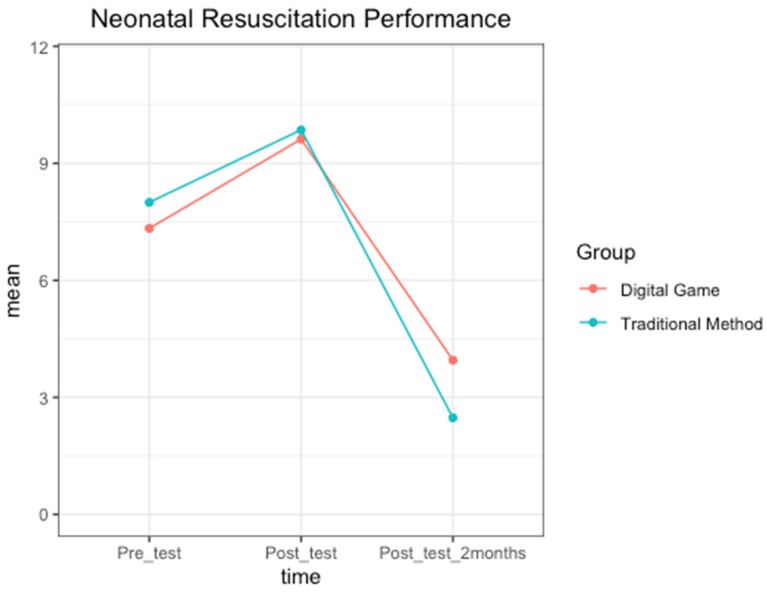
The performance of participants by condition and time point.

The pattern of results is also depicted in Figure 3, showing a line graph plotting the participant neonatal performance with the time variable on the *x*-axis. 

**Figure 4:**
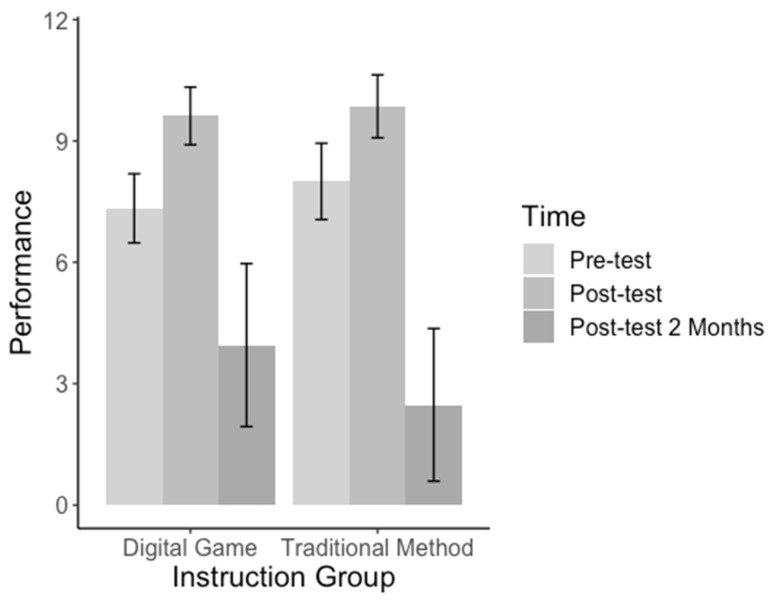
The performance of participants at different time points grouped by instruction method.

Figure 4 shows a bar graph plotting the participant neonatal performance (*y*-axis) across instruction methods (*x*-axis), with error bars representing ± one standard error. This graph shows that, in both conditions, participant performance increased significantly from the *Pre-test* to the *Post-test*, and it also decreased significantly from both the *Pre-test* and the *Post-test* to the *Post-test after 2 Months*.

### 4.3. Multiple Pairwise Comparisons

Given the main effect of time, multiple pairwise comparisons were conducted to determine if the performance change was simply due to the time factor. Specifically, multiple paired *t*-test analyses for the *Time* variable were carried out, ignoring the group, with a Bonferroni correction for the *p*-values. The results showed that all pairwise comparisons were statistically significant, as illustrated in Table 5. Findings revealed a significant increase in performance from the *Pre-test* to the *Post-test*. In contrast, the findings showed a significant decrease from both the *Pre-test* and the *Post-test* to the *Post-test after 2 Months*.

## 5. Discussion, Contributions, and Limitations

This section discusses the results of this experimental study, its limitations, and directions for future research.

### 5.1. What Were Participants’ Perceptions of the RETAIN Digital Game Simulator?

The results show that, on average, participants enjoyed playing and learning within the RETAIN digital game simulator, which they found useful to refresh their neonatal resuscitation knowledge. One explanation for this result is the interactive and non-linear nature of the RETAIN digital game simulator that makes the player feel in control [8]. Specifically, according to self-determination theory [10], when players feel a sense of autonomy, competence, and relatedness, their intrinsic motivation for the task is better supported. Enjoyment is believed to be derived from one’s intrinsic motivation. It is believed that enjoyment is derived from these three needs being met [11].

These results are aligned with the results of other relevant studies in the related literature. For example, a similar result regarding the overall positive attitudes toward the RETAIN digital game simulator was found in a sample of paramedics [16]. Also, an experimental study that randomly assigned medical students to one of two conditions (i.e., training their neonatal resuscitation skills using a computerized simulator versus a lecture video) found that participants were very satisfied with training in the computerized environment [25].

One of the limitations of this study is that data were only collected regarding the participants’ attitudes toward the treatment and not the control (traditional) intervention (i.e., the lecture video), given the participants’ limited time. Future work will collect attitudinal data toward both media, comparing participant attitudes and exploring any influence of their attitudes on learning outcomes, especially as a recent systematic review revealed that, in general, medical simulations seem to motivate HCPs [9]. Additionally, a literature review of neonatal resuscitation simulators revealed that participants preferred high-fidelity simulators over traditional ones across all the studies reviewed [29].

### 5.2. Is There an Interaction between the Instructional Method and Time on Participants’ Neonatal Resuscitation Performance?

First, the present study found that participants’ performance significantly improved from the pre-test to the first post-test, regardless of instructional condition. This means that RETAIN was at least as good as a more traditional approach (i.e., video lecture) in refreshing HCPs’ neonatal resuscitation knowledge. Other research has found that medical simulations tend to improve HCP performance [9]. Regarding the traditional method, the performance increase is an expected result, as prior research found that standard NRP training can improve trainees’ resuscitation knowledge and skills [20,21,22,23]. Regarding the digital game simulator, other researchers found that a computer-based simulator also improved performance; moreover, there was no statistically significant difference between the computer-based simulator and a seminar-based instructional approach [24].

Also, results show that the pattern of results was the same across the two conditions. Indeed, another experimental study found no performance difference between neonatal resuscitation training with a high-fidelity simulator versus traditional low-fidelity equipment, and no difference in non-technical skills and stress levels [30]. These findings from the related literature are in line with our present study whose findings indicate that, while there was a statistically significant change over time (i.e., main effect of time), this change did not differ between the conditions (i.e., non-significant interaction between time and condition). A similar study conducted on a sample of paramedics showed a similar pattern of results, suggesting that the RETAIN digital game simulator approach is generalizable and reliable [16].

Second, the present study found a significant decrease in performance after delayed testing (i.e., two months). The existing literature shows that neonatal resuscitation knowledge and performance of critical skills decrease over time, with skills decaying more than knowledge [20]. Thus, the results obtained in the present experiment (i.e., the decline in performance after two months) reflect a natural decline that may occur regardless of the training method in the absence of frequent training. Indeed, the pattern of results has been consistent regardless of the training method, suggesting that the loss of performance occurred naturally. These results were echoed in the related literature. An experimental study comparing two instructional methods (computerized simulator vs. lecture video) also found that third-year medical students’ knowledge decreased after four and eight months for both conditions, even after receiving booster training [25]. They also found no skill difference between the two conditions after eight months, compared to after two months in the present study. Similarly, they found that the computerized simulator and the lecture videos were as effective in maintaining neonatal resuscitation skills. For example, aligned with the results of the current experiment, McCaw and colleagues [26] found skill decay at 2 months and 4 months following nursing students’ initial training (tele-simulation via a remote video vs. in-person resuscitation training). Comparable to the results of the current experimental study, they did not find statistical differences in neonatal resuscitation skills between the students trained in-person versus the students trained via remote videos.

Taken together, these results suggest that digital game simulators can complement more traditional training and assessment methods. Moreover, they can even be used interchangeably with such methods, as the RETAIN digital game simulator seems to be comparable in its effectiveness of boosting, retaining, and maintaining neonatal resuscitation performance to standard, more traditional methods. These tools are also more readily available than more conventional simulations that often require a dedicated physical space and materials that trainees could use in that space. Thus, HCPs can use digital games for training, which they can access anytime, anywhere, while still reaping the benefits that both digital games and more traditional methods provide for refreshing participants’ neonatal resuscitation knowledge.

Another limitation of this experimental study is its small sample size. However, such populations are notoriously difficult to engage in such studies, given their busy and unpredictable schedule, as well as their limited free time. Future research should aim to collect more data for this population. Meanwhile, these results confirm previous findings sampling paramedics [16].

Finally, the practice of skills like PPV, intubation, umbilical venous catheterization, and chest compressions using a manikin elicits a different skill set than simulations like RETAIN or watching a lecture video. More complex trials need to be developed to further address these differences.

## 6. Contributions and Implications

The findings indicate that digital game simulators can be used to refresh participants’ neonatal resuscitation performance either as alternatives to traditional simulations or alongside them. Therefore, the results of this study suggest that more research is needed to investigate novel instructional methods, such as digital games that simulate real-world scenarios in the delivery room in a risk-free manner. Results of a meta-analysis showed that simulation-based medical education outperforms traditional approaches to clinical teaching [36]. This suggests that more refinements are necessary to the design of the RETAIN digital game simulator to achieve better performance with respect to more traditional approaches to neonatal resuscitation training and maintenance.

Thus, these findings have implications for the design and development of alternative training simulations in the context of neonatal resuscitation. Results suggest that digital game simulators such as RETAIN are enjoyable alternatives to more conventional booster methods, as they provide the same level of neonatal resuscitation performance retention and maintenance over time while also eliciting favourable user attitudes toward them. The decline in performance after two months from the initial booster has implications for spacing out neonatal resuscitation training sessions. The findings suggest that HCPs may require refreshers of their neonatal resuscitation knowledge more frequently than every two months. Future research will aim to determine the optimal frequency of retraining for labour and delivery room HCPs to ensure that they maintain their neonatal resuscitation knowledge and skills.

Finally, it is important to emphasize that RETAIN is not meant to replace instructor-led training and assessment using manikins, as these are still needed for practice and for skill demonstration. Instead, RETAIN is meant to be used as a frequent refresher and exposure to uncommon neonatal resuscitation scenarios, which is not possible in physical settings.

## 7. Conclusions

Labour and delivery room healthcare providers in both groups (digital game simulator treatment or video lecture control) of this randomized controlled simulation trial significantly improved their neonatal resuscitation performance immediately following the intervention. Moreover, there were no differences between the groups before or after the intervention, showing that RETAIN is comparable to a more traditional training approach, being at least as good in helping HCPs improve their performance. However, in terms of maintenance of neonatal resuscitation performance, both groups performed significantly worse on a post-test administered two months after the intervention compared to the previous two time points. Overall, the current study findings suggest that technology-rich environments such as digital game simulators can constitute attractive alternatives to traditional refresher methods used to boost and maintain healthcare providers’ neonatal resuscitation knowledge.

## Figures and Tables

**Table 1:** Descriptive statistics of the 5-point Likert-scale game-related survey items.

Digital Game Group (*n* = 21)	Mean (SD)	Range
Realistic Scenario	3.86 (0.91)	1–5
Appropriate Game Length and Pacing	3.76 (1.00)	1–5
Enjoyed Learning in this Game Format	3.29 (1.15)	1–5
Enjoyed Playing the Game	3.48 (1.17)	1–5
Game was Useful to Teach NRP Algorithm	3.67 (1.11)	1–5
Game Allowed Appropriate Decisions	3.67 (0.97)	1–5

Note: SD = standard deviation.

**Table 2:** Descriptive statistics of the neonatal resuscitation performance variables.

Intervention (*n* = 21)	Pre-Test Mean (SD; IQR)	Post-Test Mean (SD; IQR)	Post-Test-2-Months Mean (SD; IQR)
Lecture Video	8.00 (2.07; 2)	9.86 (1.71; 2)	2.48 (4.14; 6)
Digital Game	7.33 (1.88; 3)	9.62 (1.56; 2)	3.95 (4.42; 9)

Note: SD = standard deviation; IQR = interquartile range.

**Table 3:** The results of the robust two-way mixed ANOVA with 20% trimmed means.

Effect	*df*_1_	*df*_2_	*F*	*p*
Group	1	23.44	0.29	0.60
Time	2	20.56	33.13 ***	<0.001
Group × Time	2	20.56	1.28	0.30

Note: *** *p* < 0.001. The bold font indicates statistical significance.

**Table 4:** The results of the robust two-way mixed ANOVA.

Function	Effect	Estimate	*p*
*Sppbb*	Time (Pre–Post)	−2.171 ***	**<0.001**
	Time (Pre–Post2M)	5.368	
	Time (Post–Post2M)	9.621	
*Sppba*	Group (Digital–Traditional)	−0.5881	0.484
*Sppbi*	Interaction (Pre–Post × Digital–Traditional)	−0.5816	0.474
	Interaction (Pre–Post2M × Digital–Traditional)	−4.3690	
	Interaction (Post–Post2M × Digital–Traditional)	−2.9167	

Note: *** *p* < 0.001; Pre = Pre-test; Post = Post-test; Post2M = Post-test after 2 Months. The bold font indicates a statistically significant time difference.

**Table 5:** The results of the pairwise comparisons using a Bonferroni adjustment for the *p*-values.

Variable 1 (*n* = 42)	Variable 2 (*n* = 42)	*t*(41)	Estimated M_D_	Cohen’s *d*
Pre-test	Post-test	−7.76 ***	−2.07	−1.14 (large)
Pre-test	Post-test After 2 Months	5.69 ***	4.45	1.33 (large)
Post-test	Post-test After 2 Months	8.21 ***	6.52	2.01 (large)

Note: *** *p* < 0.001; M_D_ = mean difference. The bold font indicates statistical significance.

## Data Availability

The data presented in this study are available on request from the corresponding author. The data are not publicly available due to privacy or ethical restrictions.

## Appendix A

The simulation scenario used for the three tests (pre-test, post-test, and post-test after two months) is detailed in this section.

Simulation Scenario for the Pre-test: There is a 26-year-old woman G1P0 with no prenatal care currently at 34 weeks pregnant. The mother received two doses of Betamethasone and MgSO_4_ prior to caesarean section due to foetal heart abnormalities.

The baby is delivered.

What are the initial assessments and steps you and your team would perform?

____________________________________________

The baby is transferred to the resuscitation table.

What are the next steps?

____________________________________________

After your initial assessment, you determine the following: heart rate (HR) 80 beats/min, blue skin colour, and apnoeic.

What are the next steps?

____________________________________________

Your next assessment: heart rate (HR) 65/min, blue skin colour, and still apnoeic.

What are the next steps?

____________________________________________

After reassessment: HR > 100/min, pink but still apnoeic.

What are the next steps?

____________________________________________

After reassessment: HR > 120/min, spontaneous breathing with increased work of breathing and grunting.

What are your final steps?

____________________________________________

No further actions are required thereafter.

## Appendix B

Table A1 shows the results of a normality assumption test for the neonatal performance measures at the three time points.

**Table A1:** The results of the Shapiro–Wilk normality test.

Variable	Lecture Video		Digital Game	
	Shapiro–Wilk	*p*	Shapiro–Wilk	*p*
Pre-test	**0.88 ***	0.02	0.93	0.14
Post-test	**0.86 ****	<0.01	0.92	0.11
Post-test after 2 Months	**0.63 *****	<0.001	**0.76 *****	<0.001

Note: * *p* < 0.05; ** *p* < 0.01; *** *p* < 0.001. The bold font indicates statistical significance.

Moreover, there was no Mauchly’s test for the *Group* variable, as the *Group* variable had only two levels. However, the results of the Mauchly’s tests for *Time* and the *Group* × *Time* interaction are shown in Table A2, together with the sphericity corrections.

**Table A2:** The results of the Mauchly’s W test for sphericity and the sphericity corrections.

Effect	*W*	*p*	*GG_ε_*	*p_GG_*	*HF_ε_*	*p_HF_*
Time	**0.30** ***	<0.001	**0.59** ***	<0.001	**0.60** ***	<0.001
Group × Time	**0.30** ***	<0.001	0.59	0.23	0.60	0.23

Note: *** *p* <0.001; *GG_ε_* = Greenhouse–Geisser epsilon; *p_GG_* = *p*-value after the GG correction; *HF_ε_* = Huynh–Feldt epsilon; *p_HF_* = *p*-value after the HF correction. The bold font indicates statistical significance.

Findings of the two-way mixed ANOVA with regular means revealed no time × group interaction predicting participants’ neonatal resuscitation performance. However, Table A3 showed a significant large main effect of time [*F*(1.18, 47.14) = 51.32, *p* < 0.001, *η_p_*^2^ = 0.56] on the neonatal resuscitation performance.

**Table A3:** The results of the two-way mixed ANOVA with regular means.

Effect	*df*_1_	*df*_2_	*F*	*p*	*η_p_* ^2^
Group	1	40	0.17	0.68	0.004
Time	1.18	47.14	**51.32 *****	<0.001	0.562 (large)
Group × Time	1.18	47.14	1.48	0.23	0.036

Note: *** *p* < 0.001. The bold font indicates statistical significance.
